# Recorded gestational violence and neonatal outcomes in a cohort of deliveries requiring neonatal hospitalization: a 15-year hospital-based perinatal registry study

**DOI:** 10.3389/fgwh.2026.1920014

**Published:** 2026-08-26

**Authors:** Santiago Vasco-Morales, Mercy Rosero-Quintana, Alexandra Amaguaya-Guala, Emily Tamayo-Saenz, Paola Toapanta-Pinta

**Affiliations:** 1 Facultad de Ciencias Médicas, Universidad Central del Ecuador, Quito, Ecuador; 2 Servicio de Neonatología, Hospital Gineco-Obstétrico Isidro Ayora, Quito, Ecuador; 3 Grupo de Investigación en Salud Infantil y Perinatal (ISIP), Dirección de Investigación, Universidad Central del Ecuador, Quito, Ecuador

**Keywords:** administrative systems, Bayes theorem, information systems, logistic models for missing indicators, premature birth, substance & alcohol use, underreported, violence against women

## Abstract

**Background:**

Gestational violence is incompletely captured in routine perinatal registries, limiting prevalence estimation and etiologic interpretation. We evaluated associations between registry-recorded gestational violence, maternal characteristics, and neonatal outcomes in a selected hospital-based population.

**Methods:**

This cross-sectional study included 26,519 delivery records from the Perinatal Information System at a tertiary referral hospital in Quito, Ecuador, from January 2009 to June 2024. Eligibility was restricted to deliveries whose newborns required neonatal hospitalization. Associations were estimated using maximum-likelihood, Firth-penalized, and Bayesian logistic regression. Neonatal analyses examined 12 prespecified outcome–trimester combinations, analyzed as exploratory. Sensitivity analyses addressed behavioral co-exposures, exposure misclassification, informative missingness, calendar time, prior specification, unmeasured confounding, and third-trimester ascertainment opportunity.

**Results:**

Gestational violence was recorded in 1.19% of deliveries. The strongest maternal associations were observed for illicit drug use [Bayesian odds ratio [OR], 6.44; 95% credible interval [CrI], 3.78–10.74], passive smoking (OR, 3.56; 95% CrI, 2.62–4.79), and alcohol use (OR, 3.18; 95% CrI, 2.19–4.54). Third-trimester recorded violence showed the most consistent association with preterm birth (OR, 1.325; 95% CrI, 0.956–1.822; E-value, 1.57), corresponding to a risk difference of 6.5 percentage points (95% confidence interval, −0.8–14.2). The estimate weakened when restricted to births ≥34 weeks and when adjusted for calendar-related registry completeness, but increased modestly with adjustment for behavioral markers. These findings should not be interpreted as establishing causal associations.

**Conclusions:**

Routine perinatal registry data may be useful for hypothesis generation and for guiding clinical and epidemiologic research until stronger evidence from prospective studies using validated violence-assessment instruments becomes available.

## Introduction

Violence during pregnancy is an important exposure associated with adverse perinatal outcomes, including preterm birth and low birth weight (1, 2). Evidence from studies of intimate partner violence using dedicated instruments varies by geographic region, case definition, violence type, and ascertainment method. Systematic reviews have estimated global prevalence rates of approximately 9.2% for physical violence and 25.0% for any intimate partner violence during pregnancy (3, 4).

These estimates are not directly comparable with frequencies from routine administrative registries such as the Perinatal Information System (SIP), developed by the Pan American Health Organization and World Health Organization. The SIP records violence through dichotomous fields that do not capture severity, frequency, chronicity, specific type, or relationship to the perpetrator (5). Its definition includes physical, psychological, or sexual violence perpetrated by intimate partners, former partners, family members, or others. SIP-derived frequencies therefore represent documented violence during pregnancy rather than population prevalence.

This distinction has implications. Underdetection limits surveillance and opportunities for assessment during prenatal care. Recorded violence may coexist with limited prenatal care, substance use, and other markers of social vulnerability (6). In retrospective registry studies, however, these characteristics should not be considered diagnostic proxies or presumed causal mediators because temporal and causal relationships cannot be established from administrative data (1, 2, 7).

Administrative registries present challenges when exposure is uncommon or systematically underdetected. Sparse exposure data — reflected in infrequent covariates — may produce unstable estimates through complete or quasi-complete separation, exposure misclassification, and informative missingness. Trimester-specific SIP fields may reflect violence timing, clinical documentation, or both. Consequently, differences in gestational duration and ascertainment opportunities complicate interpretation of associations between third-trimester documentation and preterm birth.

Comparing maximum-likelihood, Firth-penalized, and Bayesian logistic regression assesses whether associations remain stable under sparse-data conditions. Sensitivity analyses addressing underreporting, missingness, and selection bias evaluate robustness without assuming that administrative records accurately capture the occurrence or timing of violence (8–11). Nevertheless, these analyses remain assumption-dependent and cannot eliminate differential detection, residual confounding, or selection bias.

Despite these limitations, registry data may identify patterns for investigation. Although administrative records cannot estimate true prevalence, establish causal effects, or support validated clinical prediction, they may reveal documented co-occurrence profiles and exploratory perinatal signals.

The primary objective was to assess the stability of associations between recorded gestational violence, maternal characteristics, and neonatal outcomes across maximum-likelihood, Firth-penalized, and Bayesian logistic regression models, with sensitivity analyses. The secondary exploratory objective was to characterize maternal co-occurrence profiles and neonatal outcomes associated with recorded violence in a hospital-based cohort restricted to deliveries whose newborns required neonatal hospitalization. Neonatal associations were interpreted as signals within a high-risk population rather than estimates generalizable to all institutional deliveries.

We hypothesized that the strongest associations would retain similar direction and magnitude across analytic approaches despite the low recorded frequency and sparse-data risk. We also evaluated their stability under plausible false-negative exposure-misclassification scenarios, interpreting findings as assumption-dependent sensitivity analyses rather than corrected estimates of the true associations.

## Methods

### Design, data source, and study population

We conducted a hospital-based cross-sectional study using administrative records from the Perinatal Information System (SIP) at Hospital Gineco-Obstétrico Isidro Ayora in Quito, Ecuador, from January 2009 through June 2024. This tertiary public hospital is a national referral center. The SIP captures maternal, obstetric, and neonatal information documented during maternal admission, delivery, and neonatal care.

The analytic sampling frame comprised institutional deliveries in which the newborn required neonatal hospitalization. This restriction reflects the extracted dataset itself, which included only such deliveries; the full institutional delivery population was not available for analysis. Of 29,457 available delivery records, 2,938 were excluded because violence information was missing in all three trimester-specific fields. Blank SIP fields were imported as missing values (NA) during extraction into R. This rule was applied throughout the study period, although missingness varied over time and was addressed in sensitivity analyses. The final analytic sample included 26,519 unique delivery records (Supplementary Figure 1).

### Exposure

The primary exposure was gestational violence recorded in the SIP, encompassing physical, psychological, or sexual violence documented during the current pregnancy. Exposure was recorded dichotomously by trimester.

For the maternal-profile analysis, a composite variable identified violence recorded during any trimester (1.19%; *n* = 315). Neonatal-outcome analyses used trimester-specific exposures: first trimester, 0.87% (*n* = 232); second trimester, 0.64% (*n* = 171); and third trimester, 0.58% (*n* = 155).

The exposure was interpreted as registry-recorded gestational violence rather than population prevalence. Available fields did not capture severity, frequency, chronicity, violence type, interview privacy, ascertainment method, or perpetrator identity. Analyses therefore evaluated aggregated recorded violence without estimating subtype- or perpetrator-specific associations. Because physical, psychological, and sexual violence may operate through different biological and social pathways, aggregation was considered an exposure-measurement limitation rather than evidence of equivalent effects.

Four neonatal outcomes were evaluated: preterm birth, defined as birth before 37 weeks of gestation; small for gestational age, as recorded in the SIP; 1-minute Apgar score <7; and exclusive breastfeeding at neonatal hospital discharge. Each outcome was analyzed according to the trimester of recorded exposure.

### Covariates

**Figure 1:**
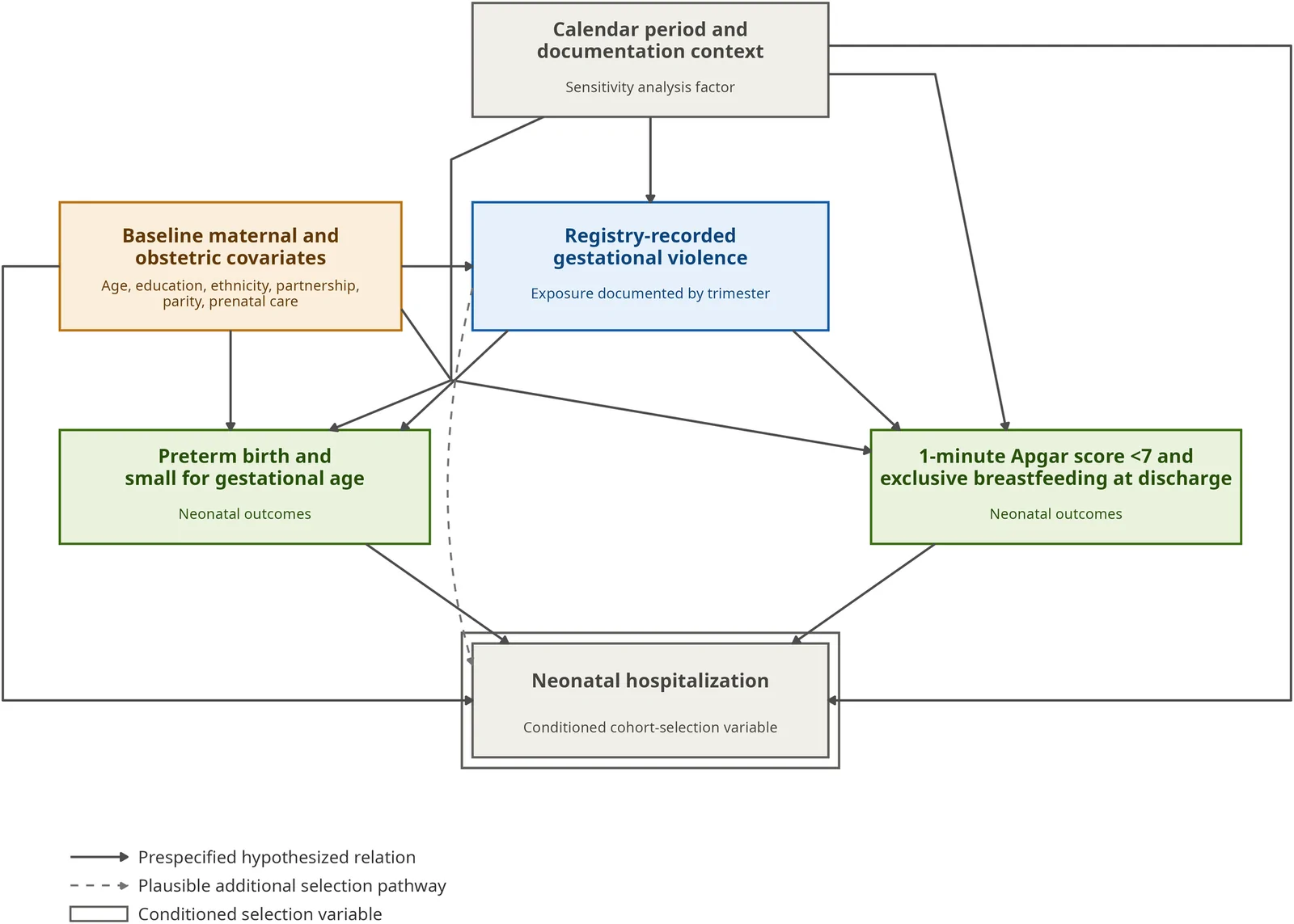
Conceptual directed acyclic graph of registry-recorded gestational violence, neonatal outcomes, and cohort selection. Baseline maternal and obstetric covariates were considered potential common causes of recorded violence, neonatal outcomes, and neonatal hospitalization. Calendar period and documentation context were represented because registry completeness and outcome occurrence varied over time. Neonatal hospitalization was a conditioned selection variable because the analytic cohort was restricted to deliveries whose newborns required hospitalization. Behavioral markers were excluded from the primary DAG because their temporal ordering relative to recorded violence could not be established; they were evaluated only in extended sensitivity models. Solid arrows represent prespecified hypothesized relations, whereas the dashed arrow represents a plausible additional selection pathway not fully captured by the measured neonatal outcomes.

Covariates were selected from the literature and an *a priori* directed acyclic graph constructed using dagitty (Figure 1). Baseline covariates included maternal age, low educational attainment, minority ethnic group, stable partnership, previous pregnancies, and prenatal care visits. Maternal age, previous pregnancies, and prenatal care visits were standardized as z-scores.

Active smoking, passive smoking, alcohol use, and illicit drug use were evaluated in the maternal-profile analysis. In neonatal-outcome analyses, alcohol use, illicit drug use, and passive smoking were included only in extended sensitivity models as concomitant behavioral markers because their temporal ordering relative to recorded violence could not be established. They were therefore not treated as *a priori* baseline confounders or formally evaluated mediators. Active smoking was excluded from neonatal-outcome models, whereas passive smoking was retained as the documented marker of tobacco exposure to limit overparameterization under sparse-data conditions.

### Statistical analysis

Analyses evaluated the stability of associations involving a low-frequency, structurally underdetected exposure. Interpretation emphasized consistency in direction and magnitude across methods and the uncertainty demonstrated by sensitivity analyses. Findings were considered epidemiological signals requiring confirmation with dedicated violence-assessment instruments rather than evidence suitable for individual clinical prediction.

Associations between maternal characteristics and violence recorded during any trimester were estimated using standard maximum-likelihood logistic regression, Firth-penalized logistic regression, and Bayesian logistic regression implemented in brms and Stan. Bayesian models used weakly informative priors: normal (0, 2.5) for regression coefficients and Student-t(3, 0, 2.5) for the intercept. Models used four Markov chains, 6,000 iterations per chain, including 2,000 warm-up iterations, with adapt_delta = 0.97 and max_treedepth = 15. Convergence was assessed using the potential scale reduction factor (R̂ < 1.01) and bulk and tail effective sample sizes (ESS ≥400).

For neonatal outcomes, 12 primary models were fitted, corresponding to each outcome–trimester combination. Each model included violence recorded during the corresponding trimester as the primary exposure. Primary models were adjusted for maternal age, low educational attainment, minority ethnic group, stable partnership, previous pregnancies, and prenatal care visits. Models that were additionally adjusted for alcohol use, illicit drug use, and passive smoking were treated as sensitivity analyses and did not define the primary estimand.

Outcome–trimester combinations were specified *a priori* to explore potential temporal windows of vulnerability. However, no single confirmatory comparison was prespecified, and no multiplicity correction was applied across the 12 models. Analyses were therefore exploratory and hypothesis-generating. The association between third-trimester recorded violence and preterm birth was interpreted as the most internally consistent neonatal signal rather than a confirmatory finding.

Eight complementary sensitivity analyses were conducted.

First, the baseline Bayesian model was compared with an extended model additionally including alcohol use, illicit drug use, and passive smoking. This analysis assessed changes after adjustment for concomitant behavioral markers and was not interpreted as formal mediation.

Second, quantitative bias analysis addressed underreporting of third-trimester violence in relation to preterm birth. The Greenland correction for exposure misclassification was applied to the crude two-by-two table (crude OR=1.536), rather than adjusted estimates, because it operates on observed cell counts:

$$A=\frac{a-(1-Sp)\times M_{1}}{Se+Sp-1}$$

$$B=\frac{b-(1-Sp)\times M_{0}}{Se+Sp-1}$$

where (a) and (b) represent observed exposed cases and noncases, (M_1) and (M_0) total cases and noncases, and (Se) and (Sp) assumed sensitivity and specificity.

Hypothetical true exposure frequencies of 3%, 5%, 8%, 10%, and 15% were evaluated. Implicit sensitivity was calculated as the ratio of the SIP-recorded frequency to each assumed true frequency, with specificity fixed at 1.0. Sensitivity uncertainty was propagated through 10,000 Monte Carlo iterations, with (Se) sampled from a uniform distribution spanning ±30% of its implicit value, following Fox, Lash, and Greenland (2005). The correction assumed nondifferential exposure misclassification. These analyses represented assumption-dependent scenarios rather than corrected estimates of the true association.

Third, exclusion of 2,938 records with missing violence information in all three trimesters was treated as potentially informative. Included and excluded records were compared using crude and weighted standardized mean differences. Inclusion probabilities were estimated using inverse probability weighting based on maternal age, low educational attainment, minority ethnic group, stable partnership, previous pregnancies, alcohol use, illicit drug use, active smoking, and prenatal care visits. Weights were truncated at the 1st and 99th percentiles, and an SMD <0.10 indicated adequate balance. The annual distribution of excluded records was examined descriptively. Because completeness varied over time, the weighting model was re-estimated using calendar periods categorized as 2009–2019, 2020, 2021–2022, and 2023–2024.

Fourth, a weighted bootstrap with 500 replicates assessed sampling variability in the association between third-trimester recorded violence and preterm birth. An E-value quantified the minimum association strength an unmeasured confounder would require with both exposure and outcome to explain the observed association.

Fifth, prior sensitivity was assessed by comparing the weakly informative normal (0, 2.5) prior with a skeptical normal (0, 0.5) prior.

Sixth, because preterm births provide less opportunity for third-trimester violence documentation than term births, analysis was restricted to births at ≥34 weeks of gestation. This restriction reduced, but did not eliminate, differences in the opportunity for third-trimester ascertainment.

Seventh, the primary model was adjusted for calendar year to account for temporal changes in documentation practices and registry completeness. Stratification was not performed because the low recorded frequency would have produced sparse cells and unstable estimates within calendar periods.

Eighth, adjusted predicted probabilities and the risk difference for third-trimester recorded violence and preterm birth were estimated through marginal standardization of the baseline-adjusted model. Bootstrap resampling with 500 replicates provided 95% confidence intervals.

### Software, reporting standards, and ethics

Analyses were performed in R version 4.5.0 using brms version 2.22.0, logistf version 1.26, WeightIt version 1.7.0, EValue version 4.1.4, dagitty version 0.3–4, and tidyverse version 2.0.0. Reproducibility was supported by setting the global random seed with set.seed (2024). Reporting followed the Strengthening the Reporting of Observational Studies in Epidemiology recommendations.

The study was approved by the Comité de Ética para la Investigación en Seres Humanos del Hospital General Docente de Calderón (CEISH-HGDC−2024-007; August 18, 2024), because Hospital Gineco-Obstétrico Isidro Ayora lacked an institutional ethics committee. Use of the institutional database was authorized by Hospital Gineco-Obstétrico Isidro Ayora under its procedures for research using anonymized secondary data. Individual informed consent was waived.

## Results

### Characteristics of the analytic cohort

The analytic cohort included 26,519 hospital deliveries. Median maternal age was 24 years (IQR, 19–31), and gestational violence was recorded in 1.19% (*n* = 315). Preterm birth was frequent, consistent with the selected population of deliveries whose newborns required hospitalization at a tertiary referral center. Maternal and neonatal characteristics are presented in Table 1.

**Table 1:** Maternal and neonatal characteristics of the analytic cohort (*n* = 26,519).

Variable	*N*	% (95% CI) or median (IQR)	Missing (%)
Maternal variables			
Maternal age	26,519	—	0
< 20 years	6,612	24.9 (24.4–25.5)	
20–34 years	16,149	60.9 (60.3–61.5)	
≥ 35 years	3,758	14.2 (13.8–14.6)	
Minority ethnic group	1,553	5.9 (5.6–6.1)	0
Stable partnership	20,029	75.5 (75.0–76.0)	0
Low educational attainment	5,427	20.5 (20.0–21.0)	0
Active smoking	402	1.52 (1.37–1.67)	12 (0.05)
Passive smoking	1,419	5.36 (5.09–5.63)	32 (0.12)
Alcohol use	741	2.80 (2.60–2.99)	7 (0.03)
Illicit drug use	144	0.54 (0.45–0.63)	0
Previous pregnancies	26,519	—	0
No previous pregnancies	10,135	38.2 (37.6–38.8)	
1–3 previous pregnancies	13,818	52.1 (51.5–52.7)	
≥ 4 previous pregnancies	2,566	9.7 (9.3–10.0)	
Prenatal care visits	26,012	—	507 (1.91)
≤ 4	6,293	24.2 (23.7–24.7)	
5–7	11,146	42.9 (42.3–43.5)	
≥ 8	8,573	33.0 (32.4–33.5)	
Mode of delivery	26,519	—	0
Cesarean delivery	13,979	52.7 (52.1–53.3)	
Vaginal delivery	12,540	47.3 (46.7–47.9)	
Neonatal variables			
Gestational age (weeks), median (IQR)	26,326	37 (35–39)	193 (0.73)
Gestational age classification	26,240	—	279 (1.05)
Preterm (<37 weeks)	9,487	36.2 (35.6–36.7)	
Term (37 + 0 to 41 + 6 weeks)	16,626	63.4 (62.8–64.0)	
Postterm (≥42 weeks)	127	0.48 (0.40–0.57)	
Birth weight (g), median (IQR)	26,513	2,650 (2,030–3,125)	6 (0.02)
Birth weight for gestational age	26,513	—	6 (0.02)
Small for gestational age (SGA; <10th percentile)	8,093	30.5 (30.0–31.1)	
Appropriate/large for gestational age (≥10th percentile)	18,420	69.5 (68.9–70.0)	
1-minute Apgar score <7	4,493	17.2 (16.8–17.7)	444 (1.67)
5-minute Apgar score <7	912	3.49 (3.27–3.72)	415 (1.57)
Male sex	14,330	54.0 (53.4–54.6)	0
Congenital anomalies	3,072	11.6 (11.2–12.0)	0
Exclusive breastfeeding at discharge	21,816	82.3 (81.8–82.7)	0

GA, gestational age; 95% CI, 95% confidence interval; IQR, interquartile range; SIP, Perinatal Information System. Values are presented as absolute frequencies and percentages with 95% CIs, or as medians with IQRs, as appropriate. The SIP gestational violence variable records dichotomous exposure (yes/no) without differentiation by type, severity, frequency, or perpetrator. The “Missing” column indicates the number and percentage of missing values for each variable; category percentages were calculated among records with valid data. All 12 outcome-trimester combinations were prespecified as exploratory, without correction for multiple comparisons; no single combination was designated as confirmatory.

### Maternal characteristics associated with recorded gestational violence

Associations between maternal characteristics and recorded violence were consistent across maximum-likelihood, Firth-penalized, and Bayesian logistic regression. The strongest positive associations involved illicit drug use, passive smoking, and alcohol use, whereas stable partnership, more prenatal care visits, and older maternal age were inversely associated with recorded violence (Table 2). Bayesian models showed adequate convergence (Supplementary Table 1).

**Table 2:** Maternal factors associated with recorded gestational violence.

Variable	Crude OR (GLM, 95% CI)	Adjusted OR (GLM, 95% CI)	Adjusted OR (Firth, 95% CI)	Bayesian OR (95% CrI)	Posterior probability	*P* value (GLM)	*P* value (Firth)
Illicit drug use	36.35 (24.51–53.00)	6.47 (3.79–10.92)	6.41 (3.77–10.80)	6.44 (3.78–10.74)	100.0%	<0.001	<0.001
Alcohol use	9.48 (7.10–12.50)	3.20 (2.19–4.59)	3.22 (2.21–4.61)	3.18 (2.19–4.54)	100.0%	<0.001	<0.001
Active smoking	11.73 (8.32–16.19)	1.18 (0.70–1.93)	1.19 (0.71–1.94)	1.18 (0.71–1.94)	73.9%	0.525	0.512
Passive smoking	7.16 (5.55–9.15)	3.58 (2.63–4.81)	3.59 (2.64–4.83)	3.56 (2.62–4.79)	100.0%	<0.001	<0.001
Previous pregnancies (z score)	1.33 (1.22–1.45)	1.53 (1.36–1.71)	1.53 (1.36–1.71)	1.53 (1.36–1.71)	100.0%	<0.001	<0.001
Prenatal care visits (z score)	0.68 (0.60–0.76)	0.83 (0.74–0.93)	0.83 (0.74–0.93)	0.83 (0.74–0.93)	99.97%	0.002	0.002
Stable partnership	0.68 (0.54–0.86)	0.68 (0.53–0.88)	0.68 (0.53–0.88)	0.69 (0.53–0.89)	99.80%	0.003	0.004
Low educational attainment	1.70 (1.34–2.16)	1.39 (1.07–1.80)	1.39 (1.07–1.81)	1.39 (1.06–1.80)	99.01%	0.014	0.014
Minority ethnic group	1.46 (0.96–2.13)	1.20 (0.77–1.79)	1.22 (0.79–1.82)	1.19 (0.76–1.76)	78.5%	0.396	0.362
Maternal age (z score)	0.93 (0.83–1.04)	0.72 (0.61–0.84)	0.72 (0.62–0.84)	0.72 (0.61–0.84)	100.0%	<0.001	<0.001

The association between maternal characteristics and recorded gestational violence was estimated using maximum-likelihood logistic regression (GLM), Firth penalized logistic regression, and Bayesian logistic regression. Crude ORs correspond to univariable models. Adjusted ORs were estimated using multivariable models that simultaneously included all covariates shown in the table. Posterior probability indicates the probability that the odds ratio lies in the observed direction of effect (OR >1 for positive associations or OR <1 for inverse associations). Continuous variables were standardized as z scores; their ORs therefore represent the relative change in odds per 1-SD increase. OR, odds ratio; CI, confidence interval; CrI, credible interval; GLM, generalized linear model; SD, standard deviation.

### Neonatal outcomes according to trimester of recorded violence

Across the 12 outcome–trimester combinations, estimates from maximum-likelihood, Firth-penalized, and Bayesian logistic regression were directionally consistent and similar in magnitude. The limited differences between standard maximum-likelihood and Firth estimates suggested that sparse-data bias and separation did not materially affect the main results.

The association between third-trimester recorded violence and preterm birth was the most internally consistent exploratory neonatal signal across estimation methods. Uncertainty intervals for all other outcome–trimester combinations included the null (Table 3). Because the cohort was restricted to deliveries whose newborns required neonatal hospitalization, findings should not be generalized to all institutional deliveries.

**Table 3:** Associations between recorded gestational violence and neonatal outcomes across statistical methods, adjusted for baseline sociodemographic and obstetric covariates.

Outcome	Trimester	n	OR (GLM, 95% CI)	OR (Firth, 95% CI)	OR (Bayes, 95% CrI)
Preterm birth	T1	25,733	0.884 (0.669–1.168)	0.886 (0.668–1.167)	0.881 (0.664–1.160)
Preterm birth	T2	25,733	1.148 (0.839–1.570)	1.150 (0.839–1.568)	1.147 (0.835–1.559)
Preterm birth	T3	25,733	1.326 (0.958–1.836)	1.327 (0.958–1.835)	1.325 (0.956–1.822)
Small for gestational age	T1	26,009	1.176 (0.885–1.564)	1.180 (0.884–1.562)	1.173 (0.881–1.551)
Small for gestational age	T2	26,009	1.321 (0.956–1.825)	1.326 (0.955–1.821)	1.314 (0.953–1.805)
Small for gestational age	T3	26,009	1.102 (0.777–1.563)	1.108 (0.776–1.560)	1.096 (0.769–1.554)
1-minute Apgar score <7	T1	25,599	1.107 (0.790–1.551)	1.116 (0.789–1.546)	1.100 (0.771–1.531)
1-minute Apgar score <7	T2	25,599	0.859 (0.562–1.312)	0.872 (0.561–1.305)	0.849 (0.547–1.276)
1-minute Apgar score <7	T3	25,599	1.061 (0.699–1.608)	1.075 (0.698–1.600)	1.047 (0.680–1.556)
Exclusive breastfeeding at discharge	T1	26,012	0.924 (0.663–1.289)	0.916 (0.665–1.291)	0.927 (0.675–1.313)
Exclusive breastfeeding at discharge	T2	26,012	1.059 (0.712–1.577)	1.046 (0.715–1.580)	1.065 (0.728–1.618)
Exclusive breastfeeding at discharge	T3	26,012	1.130 (0.739–1.729)	1.114 (0.743–1.733)	1.141 (0.756–1.784)

Models were adjusted for maternal age, low educational attainment, minority ethnic group, stable partnership, previous pregnancies, and number of prenatal care visits. Alcohol use, illicit drug use, and passive smoking were not included in the primary models because the available data did not permit determination of whether they functioned as confounders or mediators of the association between recorded violence and neonatal outcomes. Models additionally adjusted for these behavioral markers are presented in Supplementary Table 2. All 12 outcome–trimester combinations were prespecified as exploratory analyses, without correction for multiple comparisons, and no single combination was designated as confirmatory. OR, odds ratio; 95% CI, 95% confidence interval; 95% CrI, 95% credible interval; GLM, generalized linear model; T1, first trimester; T2, second trimester; T3, third-trimester.

In unadjusted analyses, preterm birth occurred in 46.5% of women with third-trimester recorded violence and 36.1% of those without recorded violence. This descriptive comparison did not account for confounding.

### Sensitivity analyses

Additional adjustment for alcohol use, illicit drug use, and passive smoking produced minimal changes in estimates for preterm birth and 1-minute Apgar score <7. Associations with small for gestational age showed moderate attenuation but remained exploratory. Fully adjusted estimates are reported in Supplementary Table 2, with relative changes summarized in Supplementary Table 3.

Quantitative bias analysis was applied to the crude association between third-trimester recorded violence and preterm birth (unadjusted OR=1.54). Corrected odds ratios ranged from 1.55 (95% simulation interval, 1.55–1.56) under an assumed true frequency of 3% to 1.66 (95% simulation interval, 1.63–1.73) under a frequency of 15%. These estimates exceeded the observed odds ratio, as expected under predominantly false-negative exposure misclassification. The association with exclusive breastfeeding remained near the null across scenarios (Supplementary Table 4). Because these analyses relied on unverifiable assumptions regarding true exposure frequency and misclassification parameters, they represent bias scenarios rather than definitive corrected estimates.

Registry completeness varied substantially over time. When calendar period was incorporated into the inclusion-weighting model, the association between third-trimester recorded violence and preterm birth was attenuated (adjusted OR=1.02; 95% CI, 0.76–1.37), although residual covariate imbalance remained (maximum SMD = 0.20). The model without calendar-period adjustment produced less attenuation (adjusted OR=1.38; Supplementary Table 5).

Adding 1-minute Apgar score <7 as a neonatal severity indicator did not materially improve covariate balance (Supplementary Table 6). The non-calendar-adjusted IPW model achieved adequate balance (maximum SMD = 0.086; Supplementary Table 7).

The E-value for the baseline-adjusted association was 1.568, with a lower confidence bound of 1. This limited robustness, together with the confidence interval crossing the null, supports an exploratory interpretation. Bayesian prior sensitivity produced modest attenuation without changing the association's direction (Supplementary Table 8).

Restricting the analysis to births at ≥34 weeks excluded 3,575 records and retained 22,944. Within this restricted sample, the association between third-trimester recorded violence and preterm birth was attenuated and remained imprecisely estimated (adjusted OR=1.31; 95% CI, 0.894–1.88; *p* = 0.159).

Missingness in trimester-specific violence fields remained between 1.8% and 8.3% from 2009 through 2020, increased to 57.6% in 2021 and 62.3% in 2022, and declined to 9.2% in 2023 and 6.7% in 2024 (Supplementary Table 9). Annual recorded violence ranged from 0.15% to 2.33%, without a consistent monotonic pattern (Supplementary Table 9).

In a model additionally adjusted for calendar year, the association between third-trimester recorded violence and preterm birth remained positive but imprecise (adjusted OR=1.28; 95% CI, 0.92–1.77). Calendar year was inversely associated with preterm birth (adjusted OR=0.97 per year; 95% CI, 0.97–0.98; *p* < 0.001).

Marginal standardization yielded predicted preterm-birth probabilities of 35.8% among women without third-trimester recorded violence and 42.3% among those with recorded violence. The corresponding risk difference was 6.5 percentage points (95% CI, −0.8–14.2), and the risk ratio was 1.18.

## Discussion

In this hospital-based cohort spanning approximately 15 years, gestational violence was infrequently documented in the SIP, a finding compatible with structural underdetection but insufficient to estimate its underlying occurrence. Recorded violence was associated with illicit drug use, passive smoking, alcohol use, previous pregnancies, and fewer prenatal care visits. Among neonatal outcomes, third-trimester recorded violence showed the most consistent association with preterm birth. These findings neither establish causality nor estimate population prevalence but illustrate the potential and limitations of administrative registries for identifying patterns requiring further investigation.

The recorded frequency of gestational violence was 1.19%, substantially lower than estimates from international studies, which vary by region, case definition, violence subtype, and measurement instrument (12–18). This discrepancy is consistent with SIP underdetection, likely related to the limited sensitivity of a routine dichotomous field and the clinical context of documentation (11). Differential misclassification according to clinical contact or maternal and neonatal characteristics cannot be excluded; therefore, bias direction remains uncertain.

Quantitative bias analysis was applied to the crude association because the Greenland correction operates on unadjusted cell counts. Under hypothetical exposure-frequency scenarios of 3%–15% and specificity near 100%, implied registry sensitivity was low. Corrected estimates for third-trimester recorded violence and preterm birth exceeded the observed odds ratio, consistent with attenuation under predominantly false-negative misclassification. These results represent assumption-dependent bias scenarios rather than corrected estimates of the true association.

### Associated maternal markers

Maternal characteristics should be interpreted as co-occurring clinical and social markers rather than causal determinants or diagnostic proxies for violence. Illicit drug use showed the strongest association with recorded gestational violence, consistent with evidence of co-occurrence between interpersonal violence and substance use during pregnancy (19, 20). Women reporting illicit drug use had approximately sixfold higher adjusted odds of recorded violence. Although causal direction cannot be established, this pattern may support consideration of confidential, trauma-informed psychosocial assessment when clinically appropriate.

Alcohol use and passive smoking were also associated with recorded violence, consistent with previous studies of violence and substance exposure during pregnancy (20, 21). By contrast, the association with active smoking attenuated after multivariable adjustment, suggesting confounding by concurrent behavioral exposures. These factors may identify women with a higher probability of documented or undetected violence but should not be used diagnostically.

Previous pregnancies were positively associated with recorded violence, as reported in observational studies (22). Fewer prenatal care visits and absence of a stable partnership were also associated with recorded violence but should not be interpreted causally. They may reflect differences in healthcare access, autonomy, social support, or shared social determinants. The design cannot determine whether violence reduced prenatal care access, limited care reduced opportunities for detection, or both arose from common factors (23, 24).

### Sparse data and stability of estimates

The low recorded frequency created a risk of unstable estimation, particularly for infrequent covariates such as illicit drug use. Under sparse-data conditions, maximum-likelihood logistic regression may produce biased estimates or complete or quasi-complete separation (8, 9). Maximum-likelihood, Firth-penalized, and Bayesian logistic regression with weakly informative priors were therefore compared (10).

The three approaches produced directionally concordant estimates of similar magnitude across the 12 outcome–trimester combinations. Similar standard maximum-likelihood and Firth estimates suggest that separation and sparse-data bias did not materially determine the principal findings. However, cross-model agreement does not correct underreporting, residual confounding, or selection bias and indicates numerical stability rather than validity.

### Neonatal outcomes

The most consistent neonatal finding was the positive association between third-trimester recorded violence and preterm birth, although its magnitude was modest and several uncertainty intervals included the null. The direction is consistent with literature on intimate partner violence and preterm birth (25, 26). However, those studies used different populations, exposure definitions, and validated instruments; directional agreement neither establishes causal convergence nor validates the observed magnitude.

Several explanations remain plausible. The association may reflect a late gestational vulnerability window but may also result from greater documentation opportunity near delivery, accumulated social risk, differences in prenatal care, calendar-period effects, or selection conditioned on neonatal hospitalization.

Restriction to births at ≥34 weeks attenuated the association and yielded an interval including the null, a pattern compatible with—but not proof of—differential third-trimester documentation opportunity. The E-value was low, with a lower confidence bound of 1, indicating limited robustness to unmeasured confounding. The risk difference was 6.5 percentage points (95% CI, −0.8–14.2), also compatible with no difference. Moreover, calendar-year adjustment in the inverse-probability-weighting model markedly attenuated the association, suggesting that temporal variation in registry completeness, particularly during 2021–2022, may partly explain the finding.

Collectively, these results support a hypothesis-generating rather than confirmatory interpretation. Prospective evaluation would require validated violence instruments, improved temporal ascertainment, and a sampling frame not conditioned on neonatal hospitalization.

### Limitations and strengths

The principal limitations arise from the retrospective design and administrative registry. Violence was recorded through a dichotomous SIP field aggregating physical, psychological, and sexual violence without information on severity, frequency, chronicity, ascertainment conditions, or perpetrator. Because these subtypes may operate through different biological and social pathways, aggregation may have obscured subtype-specific associations. The recorded trimester may also represent documentation timing rather than violence onset or persistence.

The restricted sampling frame is a major limitation. The cohort included only deliveries whose newborns required neonatal hospitalization, explaining the high frequency of preterm birth and other complications. Conditioning on hospitalization may have introduced selection or collider bias because hospitalization can be influenced by maternal characteristics and neonatal outcomes. Estimates therefore apply only to this selected high-risk cohort and should not be generalized to all institutional deliveries.

This concern is particularly relevant for preterm birth and low Apgar scores, which commonly prompt neonatal hospitalization. Their associations may partly reflect the selection process rather than underlying relationships. Exclusive breastfeeding at discharge may likewise depend on illness severity, length of stay, and institutional practices that were incompletely captured.

The cross-sectional design precluded establishing temporality among violence, substance use, prenatal care, and neonatal outcomes. Missingness was likely nonrandom. Inverse probability weighting addressed measured differences between included and excluded records but could not eliminate bias from unobserved determinants. Completeness of violence fields was relatively stable from 2009 through 2020, worsened sharply in 2021–2022, and improved thereafter (Supplementary Table 9). Although this coincided with the COVID-19 pandemic and its aftermath in Ecuador, the underlying mechanism—clinical non-ascertainment, data-entry omission, or system disruption—could not be determined.

Quantitative bias analysis assumed perfect specificity and nondifferential misclassification. Sensitivity, in turn, was not empirically validated. Its findings therefore represent modeled scenarios rather than corrected effects and cannot exclude differential detection related to clinical contact or hospitalization.

Strengths include the large sample, long observation period, and assessment of a low-frequency exposure using complementary statistical methods. Comparison of maximum-likelihood, Firth-penalized, and Bayesian models demonstrated directional stability, while inverse probability weighting, quantitative bias analysis, weighted bootstrap, E-value estimation, and prior sensitivity analyses examined robustness to sparse data, underreporting, and selection. These methods strengthened assessment of internal consistency but did not eliminate structural measurement limitations.

### Clinical implications of imperfect registry data

Administrative registries with incomplete exposure measurement may identify patterns warranting clinical attention but cannot replace validated assessment instruments or confidential interviews. The maternal profiles identified should not be used to label, diagnose, or exclude violence.

A more appropriate implication is that co-occurring substance exposure, limited prenatal care, or reduced social support may justify sensitive, trauma-informed assessment when clinically appropriate. Registry findings may also support improvements in documentation, interviewer training, privacy safeguards, and referral pathways.

Overall, routinely collected perinatal data can generate hypothesis-generating epidemiological signals when sparse data, exposure misclassification, and selection are explicitly considered. These data do not support estimation of population prevalence or causal effects. Their principal value lies in identifying opportunities to improve ascertainment and informing prospective studies using validated instruments.

## Data Availability

The data analyzed in this study is subject to the following licenses/restrictions: The data analyzed in this study are not publicly available, as they consist of clinical records from the Perinatal Information System (SIP) containing identifiable information on obstetric patients. Use of the database was authorized by Hospital Gineco-Obstétrico Isidro Ayora under its institutional administrative procedures for research with anonymized secondary data, and the study was approved by the Health Research Ethics Committee of Hospital General Docente de Calderón (CEISH-HGDC-2024-007). Reasonable requests for data access can be directed to the corresponding author(s), subject to authorization from the relevant institution. Requests to access these datasets should be directed to snvasco@uce.edu.ec.
