# Supplementary material for: Recorded gestational violence and neonatal outcomes in a cohort of deliveries requiring neonatal hospitalization: a 15-year hospital-based perinatal registry study
Source: Front Glob Womens Health. 2026 Aug 26;7:1920014. doi: 10.3389/fgwh.2026.1920014 (PMC13553958; doi:10.3389/fgwh.2026.1920014)
Supplement: Supplementary file 2 [file Datasheet2.pdf]

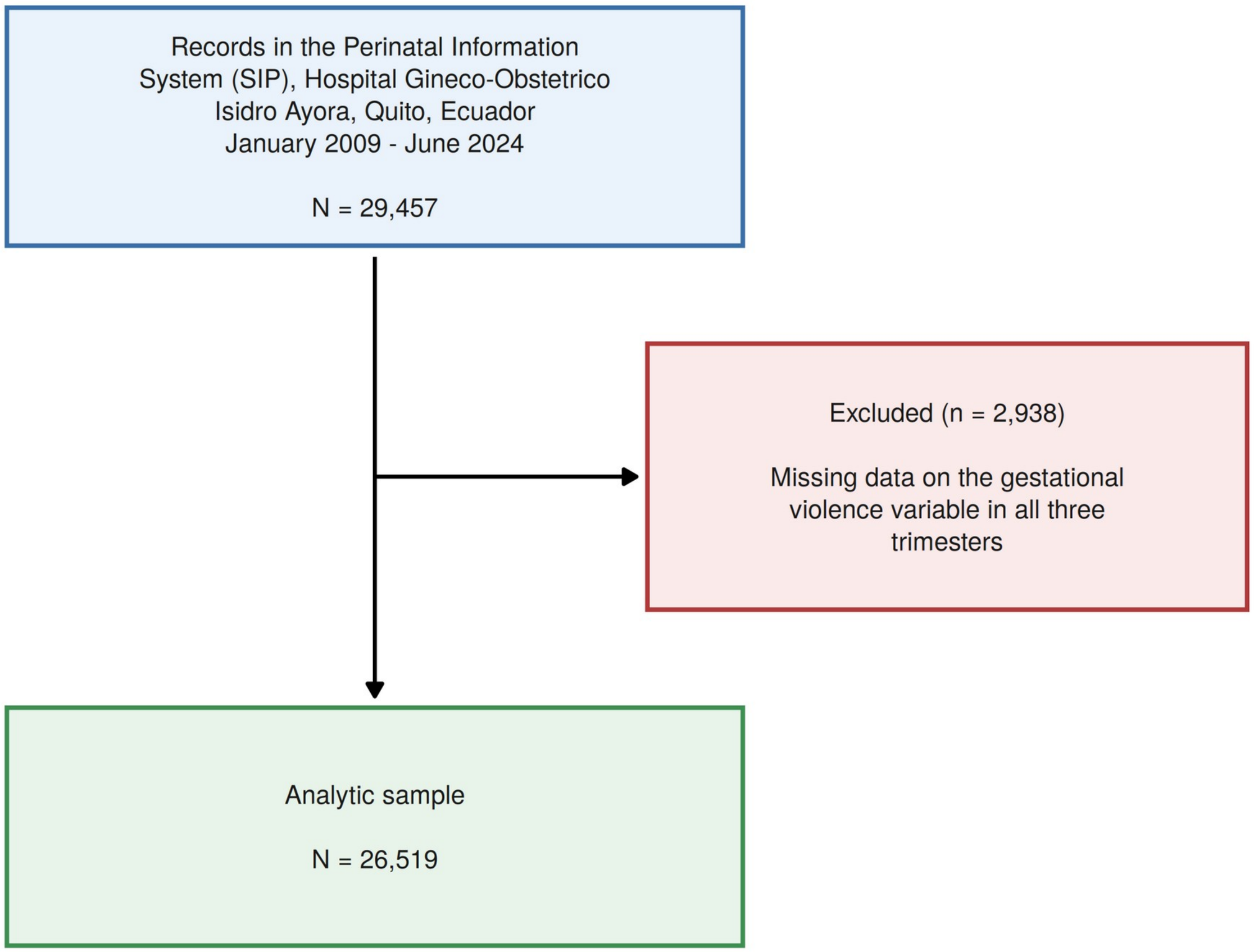

Supplementary Figure S1
STROBE flow diagram of analytic sample selection

Supplementary Table S1

Bayesian model diagnostics for the maternal profile associated with recorded gestational violence

Parameter	Posterior mean	Posterior SD	Rhat	Bulk ESS	Tail ESS
Intercept	−4.666	0.119	1.001	23,637	12,030
Illicit drug use	1.859	0.267	1.000	18,677	11,422
Alcohol use	1.155	0.187	1.000	21,114	12,504
Active smoking	0.166	0.259	1.000	18,287	13,657
Passive smoking	1.268	0.153	1.000	20,179	12,302
Previous pregnancies, z score	0.423	0.059	1.000	14,996	13,105
Prenatal care visits, z score	−0.189	0.059	1.000	21,943	12,879
Stable partnership	−0.378	0.130	1.000	23,036	11,715
Low educational attainment	0.327	0.136	1.000	21,674	12,494
Minority ethnic group	0.164	0.213	1.000	22,855	11,301
Maternal age, z score	−0.327	0.081	1.000	15,391	13,124

Posterior means and standard deviations (SDs) are presented for coefficients on the logit scale from the Bayesian logistic regression model (N = 26,519). Rhat, potential scale reduction factor; values <1.01 indicate adequate convergence. Bulk ESS, effective sample size for the bulk of the posterior distribution; tail ESS, effective sample size for the tails of the posterior distribution. All parameters reached Rhat ≤1.001 and ESS >14,000, indicating excellent convergence. Priors were normal(0, 2.5) for coefficients and Student t(3, 0, 2.5) for the intercept. Models were estimated using four chains and 6,000 iterations per chain, including 2,000 warm-up iterations, with adapt_delta = 0.97.

Supplementary Table S2

Sensitivity analysis: associations between recorded gestational violence and neonatal outcomes additionally adjusted for concomitant behavioral markers (alcohol use, illicit drug use, passive smoking)

Outcome	Trimester	n	OR (GLM, 95% CI)	OR (Firth, 95% CI)	OR (Bayes, 95% CrI)
Preterm birth	T1	25,695	0.894 (0.671–1.191)	0.896 (0.671–1.189)	0.893 (0.667–1.189)
Preterm birth	T2	25,695	1.203 (0.872–1.659)	1.204 (0.872–1.656)	1.202 (0.871–1.653)
Preterm birth	T3	25,695	1.397 (1.001–1.949)	1.397 (1.001–1.946)	1.397 (1.006–1.939)
Small for gestational age	T1	25,971	1.079 (0.804–1.448)	1.083 (0.804–1.446)	1.078 (0.797–1.446)
Small for gestational age	T2	25,971	1.235 (0.886–1.722)	1.240 (0.885–1.718)	1.233 (0.875–1.709)
Small for gestational age	T3	25,971	1.026 (0.717–1.467)	1.032 (0.717–1.463)	1.020 (0.714–1.442)
1-minute Apgar score <7	T1	25,561	1.150 (0.815–1.623)	1.160 (0.814–1.618)	1.142 (0.795–1.603)
1-minute Apgar score <7	T2	25,561	0.888 (0.577–1.367)	0.901 (0.576–1.359)	0.882 (0.559–1.334)
1-minute Apgar score <7	T3	25,561	1.103 (0.722–1.683)	1.116 (0.721–1.674)	1.090 (0.703–1.629)
Exclusive breastfeeding at discharge	T1	25,974	1.038 (0.735–1.465)	1.029 (0.738–1.467)	1.043 (0.747–1.484)
Exclusive breastfeeding at Discharge	T2	25,974	1.226 (0.812–1.852)	1.211 (0.816–1.856)	1.233 (0.828–1.906)
Exclusive breastfeeding at discharge	T3	25,974	1.300 (0.839–2.015)	1.281 (0.844–2.018)	1.310 (0.860–2.082)

This sensitivity analysis reproduces the primary models in Table 3 with an *additional adjustment for alcohol use, illicit drug use, and passive smoking, which cannot be unambiguously classified as baseline confounders or as mediators. Differences relative to Table 3 should not be interpreted as evidence of mediation. OR, odds ratio; 95% CI, 95% confidence interval; 95% CrI, 95% credible interval; GLM, generalized linear model; T1, first trimester; T2, second trimester; T3, third trimester.*

Supplementary Table S3
Exploratory attenuation analysis in Bayesian models

Outcome	Trimester	Model A OR (95% CrI)	Model B OR (95% CrI)	Ratio of ORs (95% CrI)	Relative change (%)
Preterm birth	T1	0.883 (0.669-1.166)	0.893 (0.667-1.189)	0.989 (0.659-1.485)	-1.1
Preterm birth	T2	1.169 (0.843-1.610)	1.202 (0.871-1.653)	0.970 (0.623-1.530)	-2.8
Preterm birth	T3	1.351 (0.979-1.884)	1.397 (1.006-1.939)	0.965 (0.609-1.536)	-3.4
Small for gestational age	T1	1.169 (0.878-1.550)	1.078 (0.797-1.446)	1.086 (0.716-1.651)	7.8
Small for gestational age	T2	1.339 (0.962-1.848)	1.233 (0.875-1.709)	1.086 (0.682-1.749)	7.9
Small for gestational age	T3	1.117 (0.782-1.566)	1.020 (0.714-1.442)	1.092 (0.661-1.797)	8.7
1-minute Apgar score <7	T1	1.115 (0.791-1.540)	1.142 (0.795-1.603)	0.975 (0.603-1.593)	-2.5
1-minute Apgar score <7	T2	0.863 (0.553-1.292)	0.882 (0.559-1.334)	0.976 (0.534-1.801)	-2.2
1-minute Apgar score <7	T3	1.069 (0.691-1.595)	1.090 (0.703-1.629)	0.979 (0.540-1.765)	-1.9
Exclusive breastfeeding at discharge	T1	0.913 (0.658-1.287)	1.043 (0.747-1.484)	0.875 (0.543-1.420)	-14.2
Exclusive breastfeeding at discharge	T2	1.050 (0.714-1.598)	1.233 (0.828-1.906)	0.850 (0.474-1.526)	-17.5
Exclusive breastfeeding at discharge	T3	1.119 (0.751-1.747)	1.310 (0.860-2.082)	0.853 (0.468-1.588)	-17.0

Model A was adjusted for maternal age, low educational attainment, minority ethnic group, stable partnership, previous pregnancies, and prenatal care visits. Model B additionally included alcohol use, illicit drug use, and passive smoking. Relative change was estimated from posterior differences between logit coefficients. Positive values indicate attenuation after behavioral variables were incorporated; negative values indicate a relative increase in the OR, compatible with statistical suppression or sparse-data instability. Because the number of women with recorded gestational violence was small, these analyses are exploratory. T1, first trimester; T2, second trimester; T3, third trimester; OR, odds ratio; CrI, credible interval.

Supplementary Table S4

Quantitative bias analysis for underreporting of gestational violence: preterm birth and exclusive breastfeeding in the third trimester

Outcome	Trimester	Scenario	Implicit sensitivity	Corrected OR	Monte Carlo 95% CI
Preterm birth	T3	Observed (SIP)	~100%	1.536	—
Preterm birth	T3	Assumed true prevalence: 3%	19.5%	1.553	1.548–1.563
Preterm birth	T3	Assumed true prevalence: 5%	11.7%	1.569	1.560–1.585
Preterm birth	T3	Assumed true prevalence: 8%	7.3%	1.594	1.579–1.622
Preterm birth	T3	Assumed true prevalence: 10%	5.8%	1.611	1.592–1.650
Preterm birth	T3	Assumed true prevalence: 15%	3.9%	1.661	1.627–1.731
Exclusive breastfeeding at discharge	T3	Observed (SIP)	~100%	0.978	—
Exclusive breastfeeding at discharge	T3	Assumed true prevalence: 3%	19.5%	1.023	1.023–1.023
Exclusive breastfeeding at discharge	T3	Assumed true prevalence: 5%	11.7%	1.023	1.023–1.024
Exclusive breastfeeding at discharge	T3	Assumed true prevalence: 8%	7.3%	1.024	1.023–1.025
Exclusive breastfeeding at discharge	T3	Assumed true prevalence: 10%	5.8%	1.024	1.024–1.026
Exclusive breastfeeding at discharge	T3	Assumed true prevalence: 15%	3.9%	1.026	1.025–1.028

Greenland correction for exposure misclassification due to underreporting of gestational violence, applied to the crude (unadjusted) association between recorded violence and each outcome, because this correction method operates on raw exposure-outcome cell counts rather than adjusted regression estimates. Specificity was assumed to be 1.0, equivalent to no false-positive exposure classification; this one-sided assumption was not varied. Implicit sensitivity was calculated as the ratio between the prevalence observed in the SIP and the assumed true prevalence in each scenario. Sensitivity uncertainty was propagated using Monte Carlo simulation with 10,000 iterations, sampling sensitivity from a uniform distribution spanning ±30% of the implicit sensitivity, following the probabilistic bias-analysis approach for parameters without empirical validation data (Fox, Lash, and Greenland, 2005). For preterm birth associated with third-trimester recorded gestational violence, corrected ORs increased progressively under scenarios assuming greater exposure underreporting. In contrast, the association with exclusive breastfeeding at discharge remained close to the null across all evaluated scenarios. T3, third trimester; OR, odds ratio; CI, confidence interval; SIP, Perinatal Information System.

Supplementary Table S5

Inverse probability weighting analysis by neonatal outcome and trimester

Outcome	Trimester	N	Bayesian OR (95% CrI), without IPW	GLM OR (95% CI), with IPW	p value, GLM-IPW
Preterm birth	T1	26,519	0.893 (0.667-1.189)	0.888 (0.675-1.169)	0.398
Preterm birth	T2	26,519	1.202 (0.871-1.653)	1.194 (0.879-1.624)	0.257
Preterm birth	T3	26,519	1.397 (1.006-1.939)	1.380 (1.003-1.899)	0.048
Small for gestational age	T1	26,519	1.078 (0.797-1.446)	1.079 (0.814-1.429)	0.597
Small for gestational age	T2	26,519	1.233 (0.875-1.709)	1.218 (0.886-1.674)	0.225
Small for gestational age	T3	26,519	1.020 (0.714-1.442)	1.017 (0.721-1.433)	0.925
1-minute Apgar score <7	T1	26,519	1.142 (0.795-1.603)	1.147 (0.825-1.594)	0.415
1-minute Apgar score <7	T2	26,519	0.882 (0.559-1.334)	0.895 (0.593-1.350)	0.597
1-minute Apgar score <7	T3	26,519	1.090 (0.703-1.629)	1.111 (0.741-1.664)	0.611
Exclusive breastfeeding at discharge	T1	26,519	1.043 (0.747-1.484)	1.034 (0.744-1.436)	0.843
Exclusive breastfeeding at discharge	T2	26,519	1.233 (0.828-1.906)	1.238 (0.834-1.838)	0.289
Exclusive breastfeeding at discharge	T3	26,519	1.310 (0.860-2.082)	1.304 (0.857-1.985)	0.215

IPW was estimated using nine prespecified covariates (maternal age, low educational attainment, minority ethnic group, stable partnership, previous pregnancies, alcohol use, illicit drug use, active smoking, and prenatal care visits) by fitting an inclusion model in the original database (N = 29,457) to predict the probability of having complete gestational violence records across all three trimesters (N included = 26,519; N excluded = 2,938). Neonatal outcomes were not included in the weighting model. Weights were truncated at the 1st and 99th percentiles (pretruncation range, 1.00-329.18; posttruncation range, 1.01-19.96). Covariate balance after weighting was adequate, with a maximum standardized mean difference (SMD) of 0.086 (Supplementary Table S7). For preterm birth associated with third-trimester recorded violence, the E-value was 1.646, with a lower bound of 1.058. Because completeness of violence fields varied markedly by calendar year (Supplementary Table S9), we additionally re-estimated the inclusion-weighting model for this outcome incorporating calendar year as a categorical covariate (2009–2019, 2020, 2021–2022, 2023–2024); under this model, the association attenuated substantially (adjusted OR = 1.02; 95% CI, 0.76–1.37; p = 0.891), and covariate balance remained imperfect (maximum SMD = 0.20), indicating that part of the originally observed association may reflect confounding by the period-limited increase in missing data during 2021–2022. CrI, credible interval; CI, confidence interval; GLM, generalized linear model; IPW, inverse probability weighting; OR, odds ratio; SMD, standardized mean difference; SIP, Perinatal Information System; T1, first trimester; T2, second trimester; T3, third trimester.

Supplementary Table S6

Sensitivity check: inclusion-weighting model with a neonatal severity indicator

Check	Result
SMD Apgar <7 (neonatal severity), pre-IPW	-0.08
IPW weight range with severity indicator (trimmed 1st-99th percentile)	1.01–19.8

Adding a neonatal severity indicator (Apgar score <7 at 1 minute) to the inclusion-weighting model did not materially alter covariate balance, supporting the primary inverse-probability-weighting model presented in Supplementary Table S5. SMD, standardized mean difference; IPW, inverse probability weighting.

Supplementary Table S7

Covariate balance between included and excluded records in the inverse probability weighting model

Variable	Included records, n	Excluded records, n	Crude SMD	Weighted SMD
Maternal age	26,519	2,938	-0.112	-0.086
Low educational attainment	26,519	2,938	-0.115	-0.004
Minority ethnic group	26,519	2,938	-0.236	-0.012
Stable partnership	26,519	2,938	0.351	0.024
Active smoking	26,519	2,938	0.015	-0.003
Alcohol use	26,519	2,938	0.039	0.016
Illicit drug use	26,519	2,938	-0.014	-0.027
Previous pregnancies	26,519	2,938	-0.006	-0.043
Prenatal care visits	26,519	2,938	-0.038	-0.004

Crude and weighted standardized mean differences (SMDs) were calculated between records with complete gestational violence information across all three trimesters (included, N = 26,519) and records with simultaneous absence of gestational violence information across all three trimesters (excluded, N = 2,938). Inverse probability weights were truncated at the 1st and 99th percentiles (pretruncation range, 1.00-329.18; posttruncation range, 1.01-19.96). An absolute SMD <0.10 was considered indicative of adequate balance. SMD, standardized mean difference.

Supplementary Table S8

Bayesian prior sensitivity analysis for third-trimester recorded gestational violence and preterm birth

Prior specification	Median OR	95% CrI	P(OR >1)	Rhat	ESS
Weakly informative prior - N(0, 2.5) [main analysis]	1.396	1.004-1.959	97.7%	1.001	7,224
Skeptical prior - N(0, 0.5) [stronger regularization]	1.344	0.981-1.844	96.7%	1.001	7,240

Sensitivity analysis of the Bayesian prior for the association between third-trimester recorded gestational violence and preterm birth (N = 26,519; exposed women = 155). ORs are reported as medians of the posterior distribution. P(OR >1) represents the proportion of posterior draws with OR >1. The weakly informative prior, N(0, 2.5), corresponds to the main analysis and provides minimal regularization. The skeptical prior, N(0, 0.5), applies stronger regularization toward the null. Both models used four chains and 6,000 iterations per chain, including 2,000 warm-up iterations, with adapt_delta = 0.97 and max_treedepth = 15. Rhat <=1.001 and ESS >7,000 in both models indicate adequate convergence. The reduction in the OR from 1.396 to 1.344 under the skeptical prior is consistent with expected regularization, while the direction and magnitude of the association remained broadly stable across prior specifications. OR, odds ratio; 95% CrI, 95% credible interval; ESS, effective sample size.

Supplementary Table S9

Annual distribution of recorded gestational violence and missing violence data, 2009–2024

Year	N total	% recorded violence	% missing data
2009	2,326	1.41	8.25
2010	2,375	1.35	6.69
2011	2,567	2.33	6.35
2012	2,229	1.89	2.51
2013	2,315	1.24	2.07
2014	2,213	1.16	2.62
2015	2,229	1.42	1.93
2016	2,069	1.13	1.84
2017	1,817	0.73	2.15
2018	1,476	1.06	3.86
2019	1,259	0.59	6.12
2020	1,247	0.96	8.26
2021	1,553	0.15	57.56
2022	1,293	1.23	62.34
2023	1,559	0.56	9.17
2024	930	0.81	6.67

Recorded violence frequency ranged from 0.15% to 2.33% without a consistent monotonic trend. Missingness of violence fields was low from 2009-2020 (1.8%-8.3%) but rose sharply in 2021-2022 (57.6% and 62.3%, respectively) before declining in 2023-2024 (9.2% and 6.7%). The specific cause of this period-limited increase could not be determined from the available data.
